# Sensitivity Analysis of Johnson-Cook Material Constants and Friction Coefficient Influence on Finite Element Simulation of Turning Inconel 718

**DOI:** 10.3390/ma12193121

**Published:** 2019-09-25

**Authors:** Xiaoli Qiu, Xianqiang Cheng, Penghao Dong, Huachen Peng, Yan Xing, Xin Zhou

**Affiliations:** 1 School of Mechanical Engineering, Jiulong Lake Campus, Southeast University, Nanjing 211189, China; chengxq_1994@163.com (X.C.); dongpenghao@outlook.com (P.D.); jsszphuach1992@163.com (H.P.); xingyan@seu.edu.cn (Y.X.); 2 Shenyang Liming Aero-Engine (Group) Ltd., Shenyang 110000, China; zhou525157569xin@126.com

**Keywords:** J-C constitutive model, Coulomb friction coefficient, cutting conditions, finite element model, Inconel 718

## Abstract

The Johnson-Cook (J-C) constitutive model, including five material constants (*A*, *B*, *n*, *C*, *m*), and the Coulomb friction coefficient (*μ*) are critical preprocessed data in machining simulations. Before they become reliable preprocessed data, investigating these parameters’ effect on simulation results benefits parameter-selecting. This paper aims to investigate the different influence of five settings of the J-C constitutive equation and Coulomb friction coefficient on the turning simulation results of Inconel 718 under low-high cutting conditions, including residual stress, chip morphology, cutting force and temperature. A three-dimensional (3-D) finite element model was built, meanwhile, the reliability of the model was verified by comparing the experiment with the simulation. Sensitivity analysis of J-C parameters and friction coefficient on simulation results at low-high cutting conditions was carried out by the hybrid orthogonal test. The results demonstrate that the simulation accuracy of Inconel 718 is more susceptible to strain hardening and thermal softening in the J-C constitutive model. The friction coefficient only has significant effects on axial and radial forces in the high cutting condition. The influences of the coefficient *A*, *n*, and *m* on the residual stress, chip thickness, cutting force and temperature are especially significant. As the cutting parameters increase, the effect of the three coefficients will change visibly. This paper provides direction for controlling simulation results through the adjustment of the J-C constitutive model of Inconel 718 and the friction coefficient.

## 1. Introduction

Nickel-based alloys, especially Inconel 718, are preferred materials for critical components in the industrial field due to stability at high temperatures [1]. The finite element model appears to be a useful solution to study the cutting phenomenon, owing to the expensive and challenging processing of Inconel 718. The Johnson-Cook (J-C) constitutive model and tool-to-chip friction coefficient are two kinds of critical data for machining simulation, which are necessary conditions for accurate prediction of the machining process.

In recent years, a variety of constitutive models have been proposed to describe the metal cutting process, some of which were improved [2] to more accurately depict the plastic strain of unique materials. As the J-C constitutive model’s [3] characteristics are accurate and straightforward, it is generally integrated into commercial finite element software [4].

(1) $$\;\sigma =\underset{\mathrm{Strain}\;\mathrm{hardening}}{\underset{︸}{\left(A+B\dot{\varepsilon }\right)}}\underset{\mathrm{Strain}\;\mathrm{rate}\;\mathrm{hardening}}{\underset{︸}{\left(1+C\ln\frac{\dot{\varepsilon }}{{\dot{\varepsilon }}_{0}}\right)}}\underset{\mathrm{Thermal}\;\mathrm{softening}}{\underset{︸}{\left(1-{\left[\frac{T-T_{room}}{T_{melt}-T_{room}}\right]}^{m}\right)}}$$

Equation (1) contains five parameters [5], namely the yield strength, *A*, the strain hardening coefficient, *B*, the strain hardening exponent, *n*, the strain rate hardening coefficient, *C,* and the thermal softening coefficient, *m*. Besides, ε, $\dot{\varepsilon }$, and ${\dot{\varepsilon }}_{0}$ are the strain, the strain rate and the reference strain rate respectively, and *T*, *T_melt_*, and *T_room_* orderly represent the temperature, the melting temperature and the room temperature.

In the literature, the same material has multiple sets of J-C parameters, which is not conducive to select appropriate J-C values [6]. For Inconel 718, dozens of J-C constants can be found because of different element compositions and heat treatment conditions, consequently, the stress-strain curves vary widely. Therefore, it is necessary to establish a method to identify applicable J-C values [7]. Acquiring the influence of five parameters in the J-C constitutive model on simulation results, it can guide people to choose the proper values. In this context, some researches are related.

From the results of Umbrello et al. [8], it could be qualitatively concluded that J-C parameters have a significant influence on the residual stress, chip shape, cutting force, and temperature distribution. However, further study needs to be done to investigate how J-C parameters affect the simulation results and to determine which parameters are significant factors.

Kortabarria et al. [9] identified that the coefficients *B* and *m* have a prominent effect on the residual stress profile. Nevertheless, the increments for four parameters between the selected two levels of the J-C constitutive model were different (*A*: 12%, *B*: 83%, *n*: 39%, *m*: 77%). Better controlling the increment level could make the study more convincing. When the five coefficients change in the same percentage level, the effects of coefficient *B* and *m* on the residual stress seem to alter. So far, the research on Inconel 718 mostly concentrates on relatively low cutting conditions. For Inconel 718 alloy, when the cutting speed exceeds 50 m/min, it belongs to high-speed machining [10]. At high-speed cutting conditions, the research on Inconel 718 is mostly about chip deformation, instead of the residual stress. As high-speed machining is paid more attention, the effect of high-speed machining should be explored.

The friction between the workpiece and tool has an important influence on the plasticity deformation of materials [11], and undoubtedly, it is important to affect the finite element model simulation result. Komvopoulos [12] first used the average Coulomb friction coefficient to simulate the relationship between the friction coefficient and the residual stress distribution. In the present research, the friction coefficient was determined by empirically fixed values or trial calculation. In the literature, the Coulomb friction coefficient of Inconel 718 is set in a large range, such as 0.23 [9], 0.5 [2], and 1.0 [13]. Therefore, the effect of the friction coefficient on the simulation is also worth analyzing to determine the appropriate friction coefficient and control the simulation results.

From the literature review, it can be concluded that the topics of the J-C constitutive model and the friction coefficient have been thoroughly investigated so far. The content of the research mostly focuses on the J-C model and friction factor qualitatively, which is not conducive to the selection and adjustment of J-C parameters. There are only a few articles that try to find out which parameters have a significant effect and generally consider cutting parameters located at relatively low conditions. The full application of high-speed machining makes this problem more evident and essential. It is worthwhile to study the different influence of the J-C constitutive model and friction coefficient on machining results at high cutting conditions. Besides, no study has mixed the J-C constitutive model and the friction coefficient, comparing the significance of the six coefficients. These two preprocessed data are the key factors affecting the accuracy of the simulation.

This paper aims to investigate the influence difference of five parameters in the J-C constitutive model and friction coefficient on machining-induced residual stresses, chip morphology, cutting force and temperature of turning Inconel 718 at the low-high cutting conditions with the hybrid orthogonal test. The finite element model was first verified by comparing the residual stress and chip thickness of the experimental and simulation results. Then, under the low-high cutting conditions, carrying out a 3-level 6-factors hybrid orthogonal test, the sensitivity study results were acquired by analysis of variance (ANOVA) at a 95% confidence level, which can provide direction for adjusting simulation results.

## 2. Methods 

### 2.1. Simulation Model

A three-dimensional (3-D) finite element model was used to perform the cylindrical turning simulation of Inconel 718 alloy with a hardness of 43 HRC and main chemical composition of 52.86% Ni, 19.085% Cr, 19.15% Fe, 5.085% Nb, 3.105% Mo, 0.71% Ti. The machining-induced residual stress profile, chip shape, cutting force, and temperature were investigated and compared with the experiment to verify the accuracy of the finite element method (FEM). 

The 3-D turning module of AdvantEdge v7.1 software was used for the cutting simulation. The finite element commercial software specialized for machining simulations has been used similar to the work by Huang et al. [14], Salman et al. [15] and Li [16]. The software allows a coupled mechanical and thermal modeling using an explicit integration method and provides a high-quality solution for an elastic-viscoplastic cutting simulation by adopting automatic remeshing technology to avoid the problem of severe distortion of mesh.

**Figure 1:**
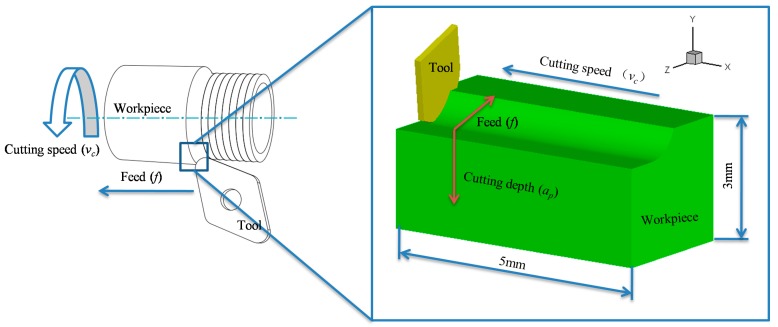
Schematic diagram of the three-dimensional (3-D) turning simulation model.

The cutting model shown in Figure 1 has been created to obtain precise cutting force, cutting heat, chip, and residual stress. In this model, the workpiece and tool were respectively modelled as an elastic-viscoplastic body and a rigid body. Simplify the workpiece and tool respectively for balancing simulation efficiency and accuracy. For the workpiece, a small part of the cutting area was selected on the workpiece, and the machining path was replaced by a straight line instead of an arc. The workpiece height was 3 mm considering the depth of cut and the influence depth of residual stress. To obtain the stable cutting result, the length of the workpiece was 5 mm (the cutting force tends to be stable at a distance of 1 mm). For the tool, the tool area was selected near the cutting edge and the main parameters of the tool insert were remained. The simulation model is very close to the real machining situation.

The cutting length was set at 6 mm in order to completely remove a layer of material and avoid stress concentration at the workpiece. When the cutting length was 5 mm, there was a large stress concentration at the tail, which caused a large error in residual stresses. The tool processed 5 mm and was in air-cut during the last 1 mm. Aiming to balance the accuracy with efficiency of the simulation, the minimum element size was set as 30 μm, and the mesh refinement depth was set as 400 μm. With the tool fixed, the cutting speed (*v_c_*) was applied in the x-direction on the workpiece. A constant coulomb friction coefficient of 0.3 was adopted to model the tool-workpiece contact, and the heat transfer coefficient was set at 20 W/m^2^∙K, considering heat exchange between the tool-workpiece and environment. The J-C constitutive constants and the material properties of the Inconel 718 alloy are detailed in Table 1 and Table 2. All the data in Table 2 were taken into account in the simulations. The elastic modulus and Poisson’s ratio are consistent with the experimental data from an X-ray diffractometer.

The chip morphology, the cutting force, and heat are easily obtained in the simulation software, and the method for getting the circumferential residual stress is detailed in Ma et al. [17].

### 2.2. FEM Validation

**Figure 2:**
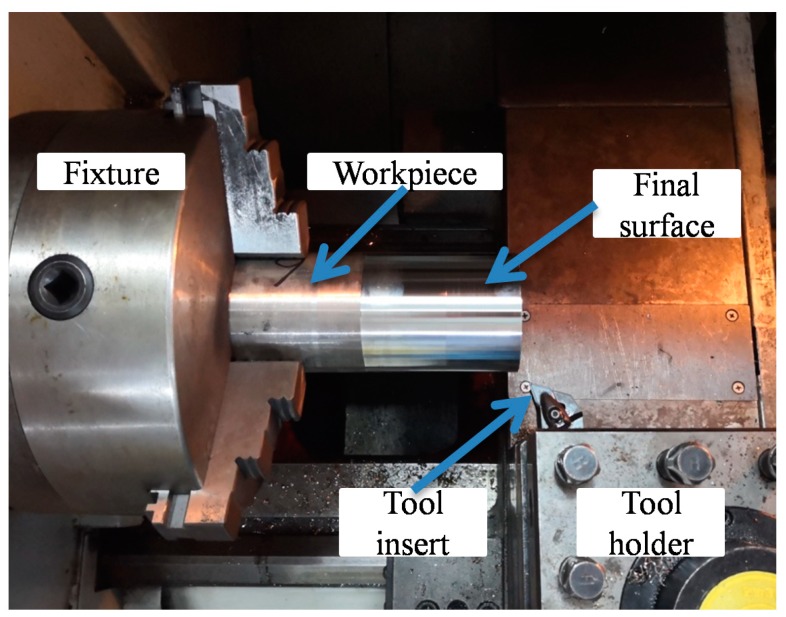
Turning experimental setup in the CNC lathe.

To validate the finite element (FE) model, low and high cutting conditions (Table 3) were selected to carry out dry turning experiments in the computer numerical control (CNC) (Figure 2). The physical vapor deposited (PVD) coated carbide (M15 grade) insert (DNMG 15 04 12-SMR 1105) was used, whose parameters are summarized in Table 4. For each cutting condition, a fresh insert was adopted. The workpiece specimen was an Inconel 718 tube of 77 mm in outer diameter and 8 mm in thickness. The workpiece surface was employed at a cutting speed (*v_c_*) of 30 m/min, a feed (*f*) of 0.1 mm/rev, and a depth of cut (*a_p_*) of 0.2 mm, to ensure the same initial surface conditions. For the subsequent measurement maneuverability of residual stress in the depth direction, the axial turning distance of each test was 50 mm. The workpieces were placed for a while after processing until it was cold to room temperature. Then, the residual stresses were measured on the machined final surface, and all the chips were collected, whose morphology was taken using a metallographic microscope.

**Figure 3:**
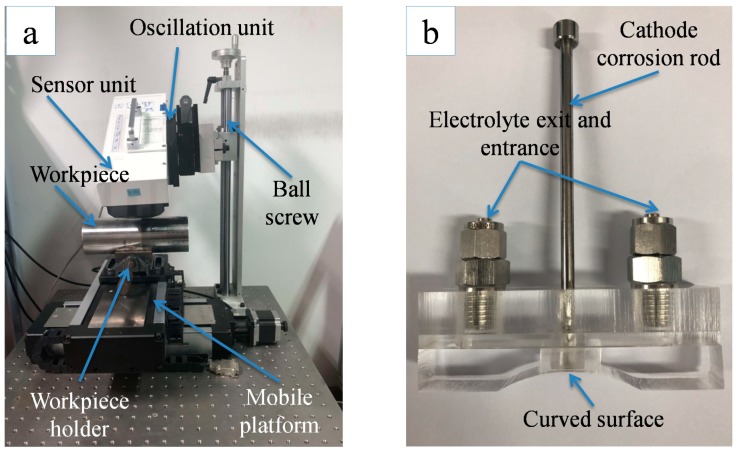
Measuring process of residual stress (**a**) X-ray diffraction (XRD) technique and (**b**) electrolyte circulation equipment for curved surface corrosion.

**Figure 4:**
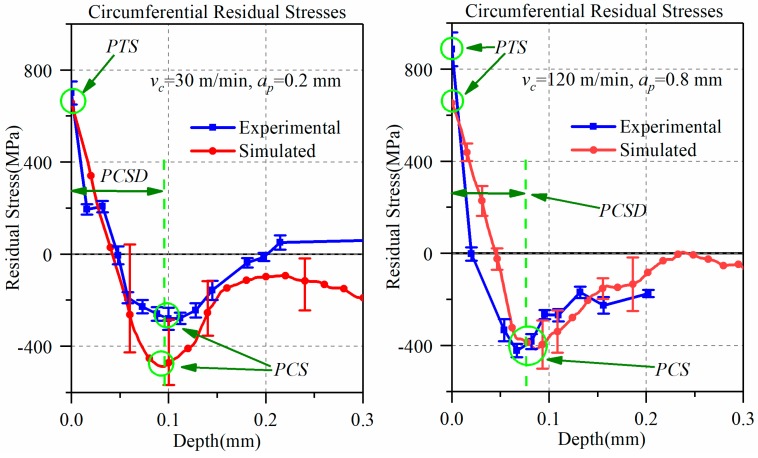
The residual stress obtained by experiment and simulation at low-high cutting conditions.

**Figure 5:**
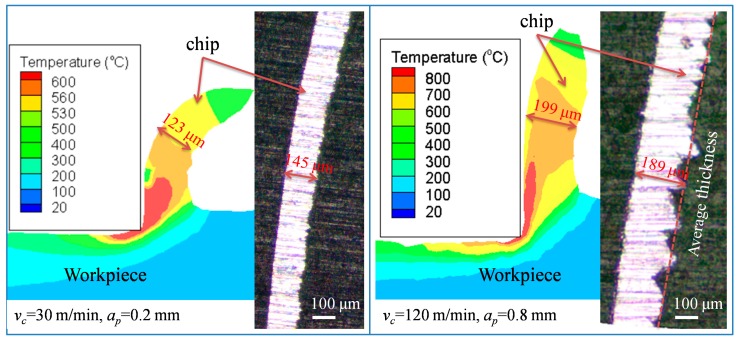
The chip thickness obtained by experiment and simulation at low-high cutting conditions.

The circumferential residual stress was measured by the X-ray diffraction (XRD) technique (Figure 3a). The measurement conditions are shown in Table 5. The residual stress in the subsurface was obtained by electrolytic corrosion and XRD. Table 6 shows the electrolytic corrosion parameters. Figure 3b is a self-made electrolyte flow device for etching a curved surface. After the corrosion rate calibration was complete, the corrosion time was fixed at 2 s every time. The stress was measured three times at each depth, and the machining surface was repeatedly corroded until the stress value was constant within ±100 MPa. The residual stress and chip thickness (*h*) obtained from experiment and simulation are compared in Figure 4 and Figure 5 at low-high cutting conditions, respectively. As shown in Figure 4, experimental residual stresses are represented by solid blue lines and error bar, which is the measurement uncertainty at each measurement depth. The simulated average stress and error bar were calculated from six locations at two appropriated sections. The measurement uncertainty in the simulation model was obtained from the maximum and minimum values of the six locations.

In the low-high cutting conditions, the residual stress distribution obtained from the experiment is highly consistent with the simulation, all of which are generally hook-shaped. In the low cutting condition, the simulation results well predict the peak tensile stress (PTS), and depth of peak compressive stress (PCSD). The peak compressive stress (PCS) is bigger than the experiment, and its error is 56%. However, the chip thickness (*h*) is 15% lower than that of the experiment. In the high cutting parameter, the simulation results are accurate for the peak compressive stress (PCS) and chip thickness (*h*). However, the peak tensile stress (PTS) of the simulation is lower, and the error is 24.5%. The depth of peak compressive stress (PCSD) is larger than the experiment, and its error is 21%. In addition, it is worth noting that the experimental chips exhibited irregular serrated chip under the high cutting condition, but no signs of serrated chip appeared in the simulation. This does not affect the research focus on chip thickness (*h*). In general, the 3-D cutting model showed higher simulation quality at the high cutting condition than that in low cutting conditions. There was less than 60% deviation of residual stresses from the modified J-C model in Arrazola et al. [2], under low cutting conditions (*v_c_* = 30 m/min, *a_p_* = 0.15 mm, *f* = 0.15 mm/rev). The simulation errors in the present paper were mostly less than 30%, and only the maximum error of peak compressive stress (PCS) was 56% under the low cutting condition. Therefore, it is certain that the simulation error of this 3-D finite element model is within the acceptable range.

### 2.3. Simulation Plan

**Figure 6:**
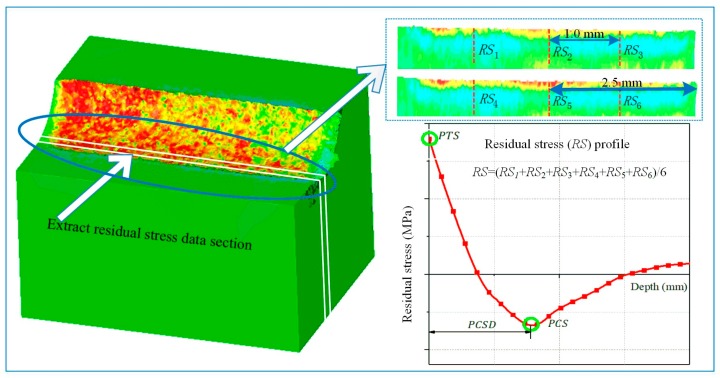
Schematic diagram of residual stress extraction from simulation results.

In order to analyze whether the preprocessed data have the same influence on the simulation results under low-high cutting conditions, three levels of each factor (five J-C parameters and friction coefficient) were simulated in FEM. The research six coefficients were arranged at the level of decreasing 20% and increasing 20% on the basis of original values, so as to carry out sensitivity analysis on finite element simulation of turning Inconel 718 in a wide range. Table 7 shows the researched J-C constitutive model parameters and friction coefficient. The hybrid orthogonal cutting test was arranged under two sets of cutting parameters, generating eighteen sets of simulation parameters. In this paper, the orthogonal test was designed based on the *L*_18_(2 × 3^6^) orthogonal table. Since there were only six experimental factors, the first column was set to the empty column (AF), and the first column did not participate in the calculation analysis. Assuming that there was no interaction between the variable factors, six factors were randomly filled into other columns to obtain mixed orthogonal test table. All the eighteen sets were performed using finite element simulation to obtain results as followed: peak tensile stress (PTS), peak compressive stress (PCS), depth of peak compressive stress (PCSD) in the cutting direction (Figure 6), average machining force (*F_c_*, *F_f_* and *F_r_*), average chip thickness (*h*), and an average value of final surface maximum temperature (*T_max_*).

## 3. Results and Discussion 

The test plan and results are shown in Table 8 and Table 9. The sensitivity analysis were obtained by ANOVA and calculated with Equation (2):

(2) $$F_{j}=\frac{S_{j}/f_{j}}{S_{e}/f_{e}}$$

where, *F_j_* is the statistic of test sensitivity, reflecting the degree of influence of each factor’s change on the index, *S_j_* is the sum of squared differences of the factors, *S_e_* is the sum of squared changes of error, *f_j_* is the degree of freedom of each factor, and *f_e_* is the degree of freedom of test error.

The sensitivity analysis of each factor to the simulation results is acquired by the value of *F_j_* in Equation (2). The test level confidence α = 0.05, and the F distribution table shows that *F*_0.05_(2,5) = 5.79. The *F_j_* of each factor is compared to 5.79 to determine the significance level. The criterion is: when *F_j_* > *F*_0.05_(2,5), the factor has a significant influence and high sensitivity, when *F_j_* < *F*_0.05_(2,5), the factor has no significant effect and low sensitivity.

### 3.1. Force and Temperature

**Figure 7:**
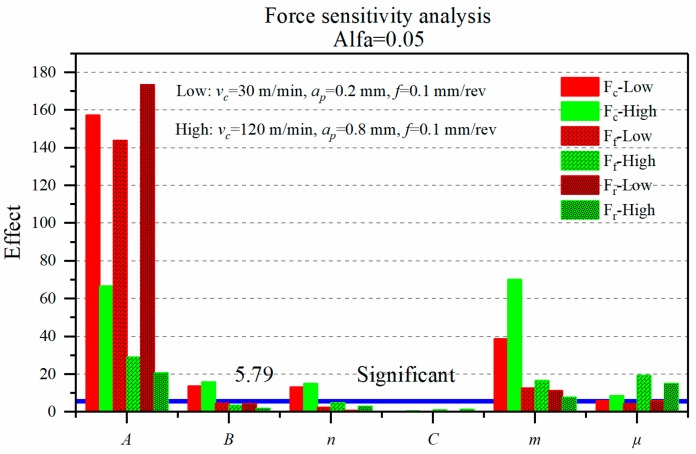
Effect of J-C parameters and friction coefficient on cutting force.

Figure 7 shows the effect of J-C parameters and friction coefficient on the three directions of the average machining force in two cutting conditions. Under the low-high cutting parameters, the coefficients that have a significant influence on the cutting force are the yield strength, *A*, the thermal softening coefficient, *m*, the friction coefficient, *μ*, the strain hardening coefficient, *B*, and the strain hardening exponent, *n*. The effects of *A* and *m* are the most considerable among six coefficients, and the strain rate hardening coefficient, *C,* has the least influence and is not significant. 

The coefficient *A* markedly affects the tangential force (*F_c_*), the axial force (*F_f_*), and the radial force (*F_r_*). The coefficient *m* shows greater influence on the tangential force (*F_c_*) than the axial force (*F_f_*) and the radial force (*F_r_*), but the coefficients *B* and *n* only have a little significant effect on the tangential force. As the cutting conditions increase, the influence of the friction coefficient (*μ*) on the cutting force gradually becomes significant. 

In addition, at different cutting conditions, the influence degree of *A* and *m* parameters on cutting force are different. At the low cutting condition, *A* is larger than *m*. Nevertheless, under high cutting conditions, the maximum reduction degree of *A* on cutting force is 88%, and the influence degree of *m* on the tangential force is increased by 81% compared with the low cutting condition. Consequently, the influence of *A* and *m* on the tangential force tends to be close. It can be confirmed that as the cutting parameters increase, the Inconel 718 material is more sensitive to the effects of thermal softening.

For the cutting force, the coefficients that have an essential influence are *A, m, μ, B,* and *n*, where *A* and *m* have the most significant effects, which also happened in the research result of Wang et al. [19], who employed a cutting speed (*v_c_*) of 500 m/min, and a depth of cut (*a_p_*) of 0.1 mm. The degree of influence of *A* and *m* on the cutting force are very disparate at low-high cutting conditions, and the friction coefficient (*μ*) only becomes significant to cutting forces at the high cutting condition. The literature [20] also mentioned that the friction coefficient should be paid attention at higher cutting conditions. 

**Figure 8:**
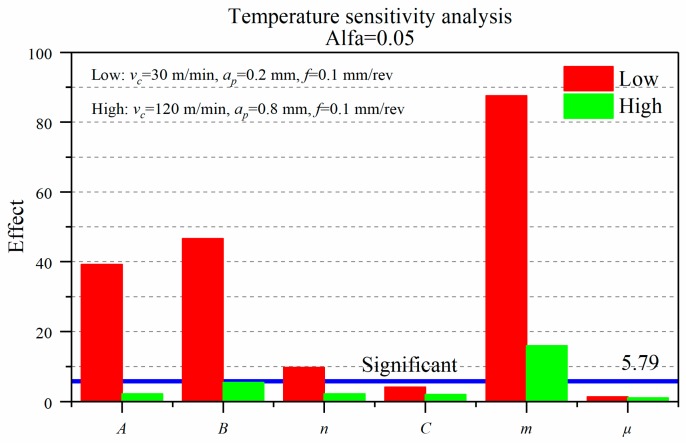
Effect of J-C parameters and friction coefficient on temperature.

Figure 8 presents the effects of J-C parameters and friction coefficient on the final surface average temperature (*T_max_*) at two cutting conditions. The factors whose influence degree from high to low at the low cutting parameter respectively are the thermal softening coefficient *m*, the strain hardening coefficient *B*, the yield strength *A*, and the strain hardening exponent *n*. The strain rate hardening coefficient *C* and the friction coefficient *μ* are less than 5.79 and not significant. In the high cutting condition, the influence of the six coefficients on the temperature is generally reduced, and the minimum reduction of effect degree in the coefficient *m* is 82% because of the heat carried by the chip principally and a fraction of heat change of the introduced workpiece. Only the coefficients *B* and *m* are significant to the temperature, and the influence degree of the coefficient *m* is still greater than *B*. 

### 3.2. Residual Stress

**Figure 9:**
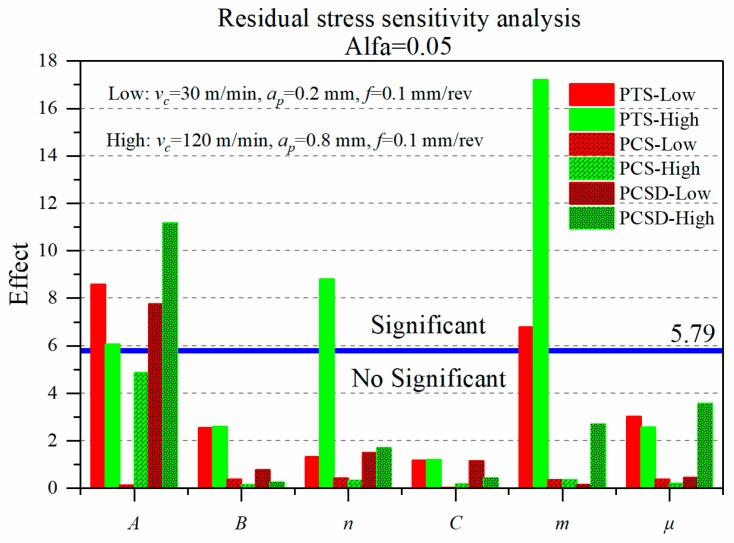
Sensitivity analysis of J-C parameters and friction coefficient on residual stress profile.

Figure 9 provides the effect of J-C parameters and friction coefficient on predicted residual stress at low-high cutting conditions. The peak tensile stress (PTS) and the depth of the peak compressive stress (PCSD) are susceptible to J-C parameters. Nevertheless, no significant coefficient was obtained to the peak compressive stress (PCS). The coefficients that both have a crucial influence on residual stress under the two sets of cutting parameters are the yield strength *A* and the thermal softening coefficient *m*. The coefficient *n* only appears to have a strong influence in the high cutting condition, and the effect of the rest of the three coefficients are less than 5.79 and not significant. 

Furthermore, the principal influence coefficients are different in the low-high cutting conditions. At the low cutting condition (*v_c_* = 30 m/min, *a_p_* = 0.2 mm), the peak tensile stress (PTS) is mainly affected by the parameters *A* and *m*, where the influence of *A* is slightly greater than *m*. The depth of the peak compressive stress (PCSD) is significantly affected by *A*. Under the high cutting condition (*v_c_* = 120 m/min, *a_p_* = 0.8 mm), the coefficients of *A*, *n,* and *m* have an important influence on the peak tensile stress (PTS), where the influence degree of *m* is more prominent than *A* and *n*. Compared with the low cutting condition, the influence factor of coefficient *A* decreases by 29.5%, and the influence factor of coefficient *m* increases by 153%. The coefficient *A* still has a significant impact on the depth of the peak compressive stress (PCSD), and the influence degree is higher than that of the low cutting condition by 44%. 

The reason why the coefficients *A* and *m* have an essential effect on the peak tensile stress (PTS) is that the surface stress is mainly affected by the mechanical and thermal load [21]. It can be seen that the coefficients *A* and *m* similarly have a remarkable influence on the cutting force and heat from Figure 7 and Figure 8. When the coefficients *A* and *m* are increased, the energy needed for material deformation increases, in consequence, the cutting force increased, and heat generation of the processing region increased. Surface tensile stress drastically changes under the effect of both the mechanical and thermal load. On account of the lower thermal conductivity of Inconel 718, cutting heat is mainly concentrated on the final surface. Therefore, the residual stress of the subsurface is primarily affected by the cutting force, resulting in the consequence that coefficient *A* appears important influence on the peak compressive stress (PCSD).

**Figure 10:**
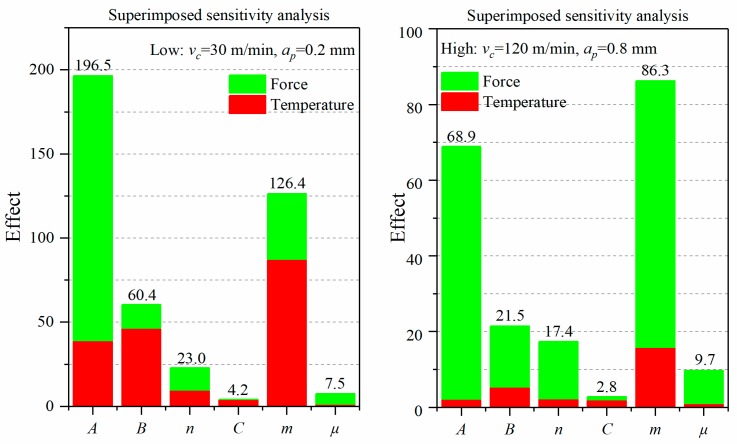
The superimposed effect of low-high cutting parameters on the tangential force and temperature.

In order to better understand the influence of mechanical and thermal load on residual surface stress, the effects of tangential force (*F_c_*) and temperature (*T_max_*) are accumulated under low-high cutting parameters. Figure 10 shows that the coefficient *A* has a larger influence on the superposition than *m* in the low cutting condition, although the effect of the coefficient *A* on temperature is obviously less than *m.* Hence, the accuracy of the yield strength *A* should be paid attention first, followed by the thermal softening coefficient *m*, with cutting simulations performed at the low cutting condition. It is necessary to execute a tensile test on the Inconel 718 alloy to obtain accurate yield strength before simulation. In the high cutting condition, the influence of the superposition is reversed. The influence of coefficients *A* and *m* on the tangential force is close. The reason why *m* has a significant influence on the maximum tensile stress (PTS) is that the effect of *m* on temperature is much higher than *A*. Therefore, the accuracy of the thermal softening coefficient *m* should be firstly focused on, followed by the yield strength *A,* when cutting simulations are performed under the high cutting condition. 

The superimposed influence of J-C parameters on tangential force and the temperature is consistent with the trend of peak tensile stress (PTS), indicating that the peak tensile stress (PTS) in the direction of cutting speed is mainly affected by the tangential force and cutting heat. Furthermore, low and high cutting conditions are mostly affected by mechanical loads and thermal loads, respectively.

For the residual stress, after adjusting the difference of variable factors to equal (±20%), compared with the research [9], the influence degree of coefficient *A* increases prodigiously, the influence factor of coefficient *B* drops to no significance, and the coefficient *m* still has a prominent effect on the residual stress profile. The influence degree will visibly change at different cutting parameters. 

### 3.3. Chip

**Figure 11:**
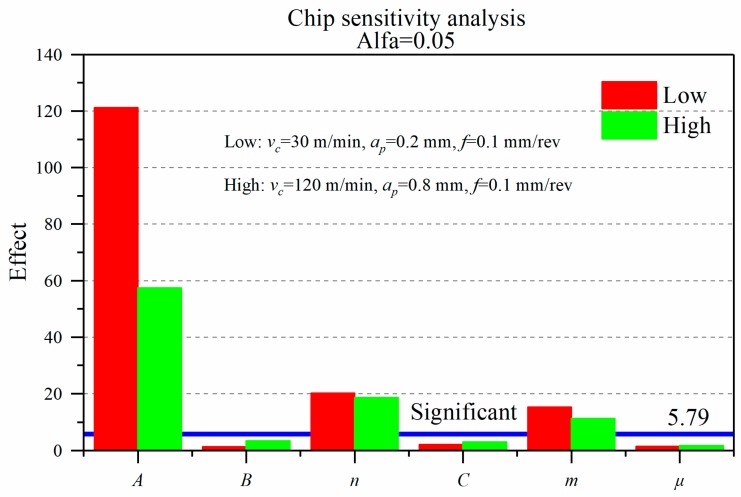
The effect of J-C parameters and friction coefficient on the chip thickness.

Figure 11 displays the sensitivity analysis of J-C parameters and friction coefficient to average chip thickness (*h*) under low-high cutting conditions. The results present that the chip thickness (*h*) is mainly affected by the yield strength *A*, strain hardening exponent *n* and the thermal softening coefficient *m*. The influence of the rest of the three coefficients on the chip thickness (*h*) are all less than 5.79 and not significant. Under the high cutting conditions, the three coefficients *A*, *n,* and *m* have the same influence tendency on the chip thickness (*h*) as in the case of under the low cutting condition. The influence degree of *A* is much larger than *n*, and *n* is slightly larger than *m*.

The chip thickness is related to the chip deformation coefficient (*λ_h_*), and the degree of deformation is principally affected by the shear slip and the extrusion and friction of the rake face. The deformation coefficient (*λ_h_*) can be calculated with Equation (3):

(3) $$\lambda_{h}=\frac{h}{h_{D}}$$

where, *h* is the chip thickness, and *h_D_* is the uncut chip thickness (equal to the feed (*f*)).

Comparing Figure 7, Figure 8 and Figure 11, the effect of the coefficients *A* and *m* on the chip thickness are consistent with the trend of the radial force (*F_r_*). The cutting force affects the change of the shear angle, which affects the deformation coefficient (*λ_h_*). As a consequence, the chip thickness is affected. The mechanism to explain the effect of the exponent *n* on the chip thickness seems to be more complicated since the impact of the coefficient *n* on the cutting force and temperature is not very significant. It needs to be further researched to comprehend this phenomenon. The coefficient *n* is consistent with the result of Ti6Al4V material in Ducobu et al. [6]. 

In the high cutting condition, the influence factor of *A* is 53% lower than that in the low cutting condition. This is coincident with the influence tendency of J-C parameters on the radial force (*F_r_*). On the one hand, this content reveals that the variation of chip thickness is mainly affected by the radial force (*F_r_*). On the other hand, this effect is greatly weakened under high cutting conditions, and the influence on the radial force (*F_r_*) is also reduced. Under this machining condition, because the deformation coefficient (*λ_h_*) of the chip generally increases, and the percentage of the heat generated by the plastic deformation increases, the Inconel 718 alloy is more sensitive to the effect of the heat softening in the high cutting condition. 

At the 95% confidence level, the friction coefficient (*μ*) universally has a lower effect on the cutting force and temperature. Therefore, it does not obviously affect residual stress and chip deformation. It is worth noting that the effect difference is close when concentrating on peak tensile stress (PTS), peak compressive stress (PCS), and chip thickness under low and high machining conditions. The disturbance from the friction coefficient is slightly affected by the cutting parameters, except the depth of the peak compressive stress (PCSD). It is well illustrated that the simulation results are mainly affected by the workpiece material (J-C) and the cutting parameters, rather than the friction coefficient.

## 4. Conclusions

In this research, a 3-D finite element turning model was established to analyze the effect of Inconel 718 alloy preprocessed data on simulation results. After finishing the sensitivity analysis of J-C parameters and friction factors to residual stress, chip thickness, cutting force and temperature, the following main conclusions can be obtained:

1. The residual stress, chip thickness, cutting force, and temperature are more sensitive to J-C parameters than the friction coefficient. The friction coefficient (*μ*) only has a significant effect on axial and radial forces under higher cutting conditions. The main influence factors of the J-C parameters are the yield strength *A*, the strain hardening exponent *n* and the thermal softening coefficient *m*. The strain rate hardening coefficient *C* hardly affects the simulation results. Turning Inconel 718 material is highly sensitive to strain hardening and thermal softening.
2. The yield strength *A* and the thermal softening coefficient *m* have an important influence on the residual stress profile. The coefficient *n* gradually becomes significant with the increase of the cutting speed and depth. The coefficients *A* and *m* appear to have a significant influence on the peak tensile stress (PTS). It is the coefficient *A* that observably affects the depth of peak compressive stress (PCSD) under both the low and high conditions, but no significant parameter is obtained for the effect on the peak compressive stress (PCS).
3. In the low cutting condition (*v_c_* = 30 m/min, *a_p_* = 0.2 mm), the influence degree of the coefficient *A* is greater than *m* on simulation results. Under the high cutting condition (*v_c_* = 120 m/min, *a_p_* = 0.8 mm), the situation is reversed. The residual surface stress is mainly affected by the cutting force in the low cutting condition. While in the high cutting condition, cutting heat plays a major role. The subsurface residual stress is always affected by the cutting force.
4. The chip is markedly affected by the yield strength *A*, the strain hardening exponent *n* and the thermal softening coefficient *m*. The influence trend invariably is *A* > *n* > *m* under low-high cutting conditions. Besides, the chip deformation is primarily affected by the radial force (*F_r_*), which are reduced in the high cutting condition.

## Figures and Tables

**Table 1:** Johnson-Cook (J-C) constitutive parameters of Inconel 718 [18].

*A* (MPa)	*B* (MPa)	*n*	*C*	*m*	${\dot{\varepsilon }}_{0}$
1290	895	0.526	0.016	1.55	0.03

**Table 2:** Physical and mechanical properties of Inconel 718 [10].

Density (kg/m^3^)	Elastic Modulus (GPa)	Poisson’s Ratio	Thermal Conductivity (W/m·K)	Specific Heat (J/kg∙K)	Thermal Expansion Coefficient (10^−6^/K)	Melting Temperature (K)
8240	214.58	0.305	10.63 (293 K)	435 (293 K)	11.8 (293 K–373 K)	1573
			14.7 (373 K)	481.4 (573 K)	13 (293 K–573 K)	
			17.8 (573 K)	514.8 (773 K)	14.1 (293 K–673 K)	
			19.6 (773 K)	573.4 (973 K)	14.8 (573 K–873 K)	

**Table 3:** Low and high cutting conditions.

	Cutting Speed *v_c_* (m/min)	Depth of Cut *a_p_* (mm)	Feed *f* (mm/rev)
Low level	30	0.2	0.1
High level	120	0.8	

**Table 4:** Tool geometry used in experimental testing and the FE simulations.

Tool Parameters	Values
Corner radius	1.191 mm
Clearance angle	6°
Rake angle	−6°
Tool lead angle	−17.5
Inclination angle	−7
Coating material	TiAlN

**Table 5:** XRD measurement conditions.

Parameters	Values
X-ray tube voltage	30.00 kV
X-ray tube current	1.20 mA
X-ray wavelength (K-Beta)	2.08480[A](Cr)
Diffraction angle (2Theta)	150.876°
Diffraction lattice angle (2Eta)	29.124°

**Table 6:** Electrolysis corrosion parameters.

Electrolytic Parameters	Values
Electrolyte	10% NaCl
Electrolyte speed	800 mL/min
Voltage	24 V
Electric current	3 A
Polishing rate	5 μm/s

**Table 7:** The researched J-C constitutive model parameters and coefficient of friction for Inconel 718.

	*A* (MPa)	*B* (MPa)	*n*	*C*	*m*	*μ*
−1 (−20%)	1032	716	0.4208	0.0128	1.24	0.24
0	1290	895	0.526	0.016	1.55	0.3
1 (+20%)	1548	1074	0.6312	0.0192	1.86	0.36

**Table 8:** Summary of FE simulation results under the low condition (*v_c_* = 30 m/min, *a_p_* = 0.2 mm). AF: empty column; PTS: peak tensile stress; PCS: peak compressive stress; PCSD: depth of peak compressive stress.

Variable Factors								Simulation Results					
Set	AF	*A* (MPa)	*B* (MPa)	*n*	*C*	*m*	*μ*	PTS (MPa)	PCS (MPa)	PCSD (μm)	*H* (μm)	*F_c_* (N)	*T_max_* (°C)
1	1	1032	716	0.4208	0.013	1.24	0.24	484	−241	102	130	129	611
2	1	1032	895	0.526	0.016	1.55	0.3	372	−197	93	138	143	691
3	1	1032	1074	0.6312	0.019	1.86	0.36	632	−272	116	155	175	818
4	1	1290	716	0.4208	0.016	1.55	0.36	397	−200	96	118	160	700
5	1	1290	895	0.526	0.019	1.86	0.24	432	−264	104	128	176	799
6	1	1290	1074	0.6312	0.013	1.24	0.3	397	−251	99	126	170	741
7	1	1548	716	0.526	0.013	1.86	0.3	518	−240	69	118	194	772
8	1	1548	895	0.6312	0.016	1.24	0.36	288	−226	94	114	185	737
9	1	1548	1074	0.4208	0.019	1.55	0.24	368	−158	78	114	181	782
10	2	1032	716	0.6312	0.019	1.55	0.3	476	−275	110	144	148	686
11	2	1032	895	0.4208	0.013	1.86	0.36	703	−293	98	137	152	717
12	2	1032	1074	0.526	0.016	1.24	0.24	494	−252	94	133	144	693
13	2	1290	716	0.526	0.019	1.24	0.36	407	−433	86	123	157	679
14	2	1290	895	0.6312	0.013	1.55	0.24	132	−247	91	127	157	708
15	2	1290	1074	0.4208	0.016	1.86	0.3	390	−313	100	120	174	799
16	2	1548	716	0.6312	0.016	1.86	0.24	442	−411	72	125	195	795
17	2	1548	895	0.4208	0.019	1.24	0.3	350	−255	93	108	169	698
18	2	1548	1074	0.526	0.013	1.55	0.36	512	−403	81	122	198	796

**Table 9:** Summary of FE simulation results under the high condition (*v_c_* = 120 m/min, *a_p_* = 0.8 mm). AF: empty column; PTS: peak tensile stress; PCS: peak compressive stress; PCSD: depth of peak compressive stress.

Variable Factors								Simulation Results					
Set	AF	*A* (MPa)	*B* (MPa)	*n*	*C*	*m*	*μ*	PTS (MPa)	PCS (MPa)	PCSD (μm)	*H* (μm)	*F_c_* (N)	*T_max_* (°C)
1	1	1032	716	0.4208	0.0128	1.24	0.24	566	−467	93	204	367	919
2	1	1032	895	0.526	0.016	1.55	0.3	687	−280	93	224	442	1031
3	1	1032	1074	0.6312	0.0192	1.86	0.36	996	−421	100	232	523	1167
4	1	1290	716	0.4208	0.016	1.55	0.36	456	−668	75	185	448	1022
5	1	1290	895	0.526	0.0192	1.86	0.24	853	−687	68	206	507	1150
6	1	1290	1074	0.6312	0.0128	1.24	0.3	510	−661	98	190	471	1069
7	1	1548	716	0.526	0.0128	1.86	0.3	961	−705	86	208	529	1103
8	1	1548	895	0.6312	0.016	1.24	0.36	393	−987	83	170	502	1078
9	1	1548	1074	0.4208	0.0192	1.55	0.24	121	−667	70	138	494	1075
10	2	1032	716	0.6312	0.0192	1.55	0.3	768	−292	99	235	437	1051
11	2	1032	895	0.4208	0.0128	1.86	0.36	858	−284	111	228	456	1070
12	2	1032	1074	0.526	0.016	1.24	0.24	666	−432	84	203	410	1041
13	2	1290	716	0.526	0.0192	1.24	0.36	586	−420	78	187	440	1000
14	2	1290	895	0.6312	0.0128	1.55	0.24	285	−620	63	194	448	955
15	2	1290	1074	0.4208	0.016	1.86	0.3	764	−519	80	187	512	1137
16	2	1548	716	0.6312	0.016	1.86	0.24	702	−558	85	198	534	1141
17	2	1548	895	0.4208	0.0192	1.24	0.3	67	−582	79	141	449	1006
18	2	1548	1074	0.526	0.0128	1.55	0.36	872	−518	68	188	540	1120
